# Supplementary figures and images for: Effect of linker on the binding free energy of stapled p53/HDM2 complex
Source: PLoS One. 2020 Apr 30;15(4):e0232613. doi: 10.1371/journal.pone.0232613 (PMC7192472; doi:10.1371/journal.pone.0232613)

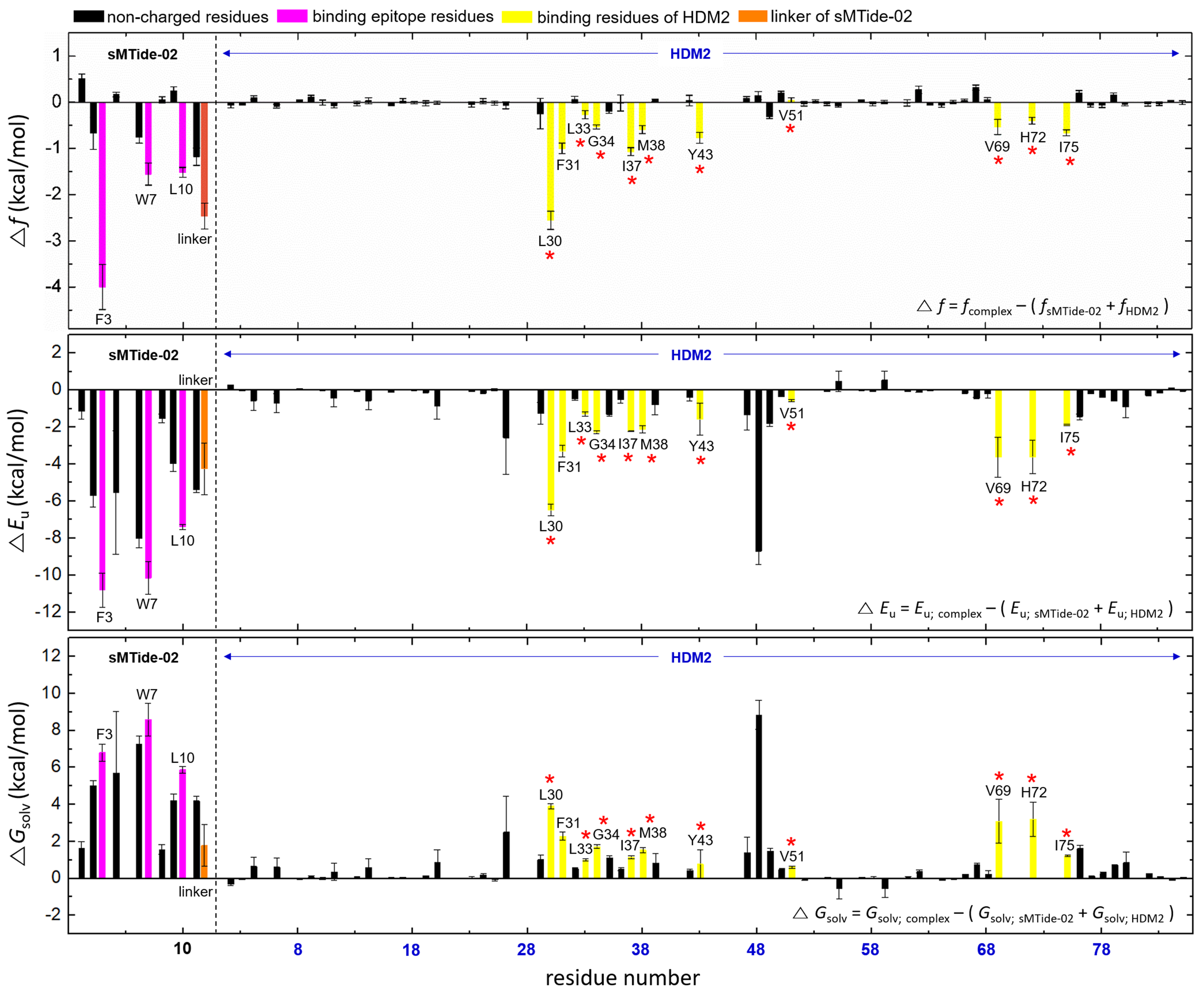

Supplement: S1 Fig — (TIF) [file pone.0232613.s001.tif]

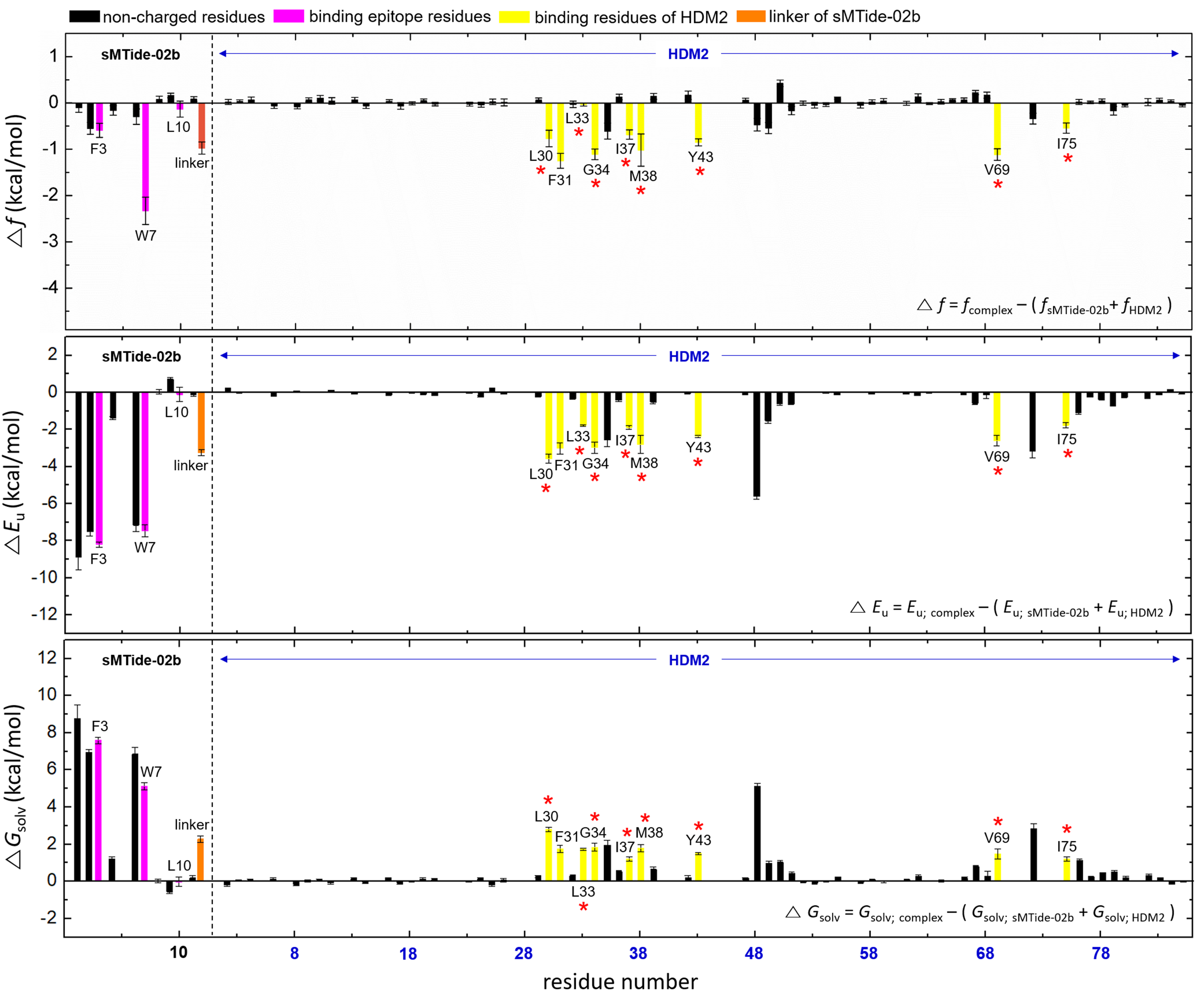

Supplement: S2 Fig — (TIF) [file pone.0232613.s002.tif]

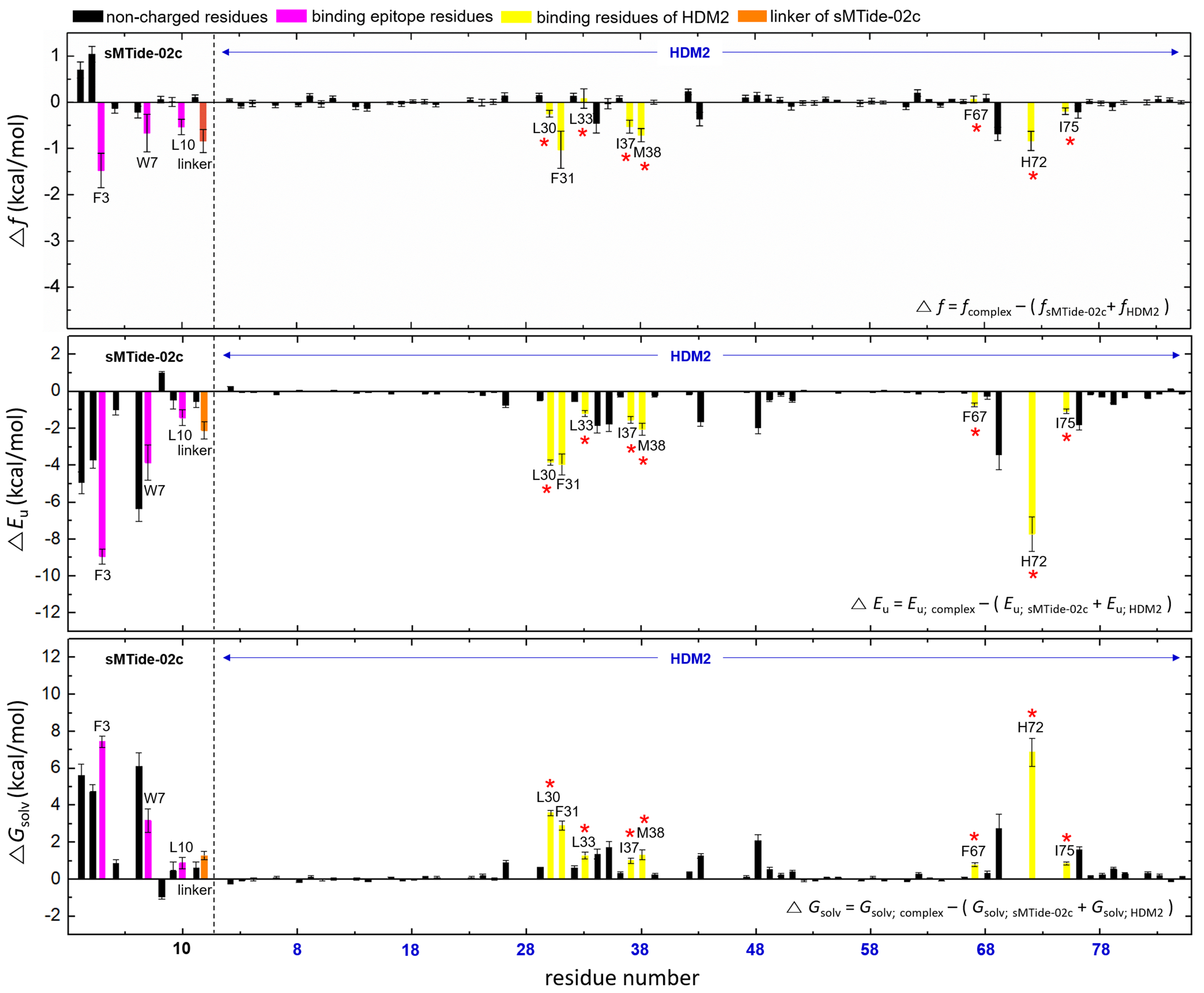

Supplement: S3 Fig — (TIF) [file pone.0232613.s003.tif]
