# Supplementary material for: Effect of linker on the binding free energy of stapled p53/HDM2 complex
Source: PLoS One. 2020 Apr 30;15(4):e0232613. doi: 10.1371/journal.pone.0232613 (PMC7192472; doi:10.1371/journal.pone.0232613)
Supplement: Attachment — Submitted filename: response_letter.pdf [file pone.0232613.s005.pdf]

## Response to Reviewers' Comments

- We thank the reviewers for their time and efforts invested in the reviewing process.
- The changes we made in the revised manuscript are marked with blue color in an annotated copy of the manuscript, which is uploaded separately.

## Peer Reviewer #1 (Review Text):

Comments:

I am unable to accept this work for publication because the study closely replicates the work of Thomas et al (Cell Cycle 2010, 9, 4560-4568). It is already well-known that staple placement is important. It is also known that the two additional staple positions the authors tried are not optimal, based on experiments carried out by Verdine et al (JACS 2007) and simulations carried out by Thomas et al. The authors also fail to discuss the differences or advantages, if any, in their approach and results with the work of Thomas et al.

Author reply:

The most distinguishing characteristic of our approach is that it enables us to decompose the effective binding energy $\Delta f = \Delta E_u + \Delta G_{solv}$, comprising the direct interaction energy ($\Delta E_u$) and the dehydration penalty ($\Delta G_{solv}$), into contributions from constituent groups. This is in contrast to the approach adopted by Thomas *et al* (more detailed in a related article, Cell Cycle 2010, 9, 1167-1181, in particular, Fig. 7) in which only $\Delta E_u$ is decomposed into contributions from constituent groups. The difference is drastic. As an illustration, we show below the residue decomposition of $\Delta E_u$ (upper panel) and $\Delta f$ (lower panel) for the stapled peptide (sMTide-02) studied in our manuscript:

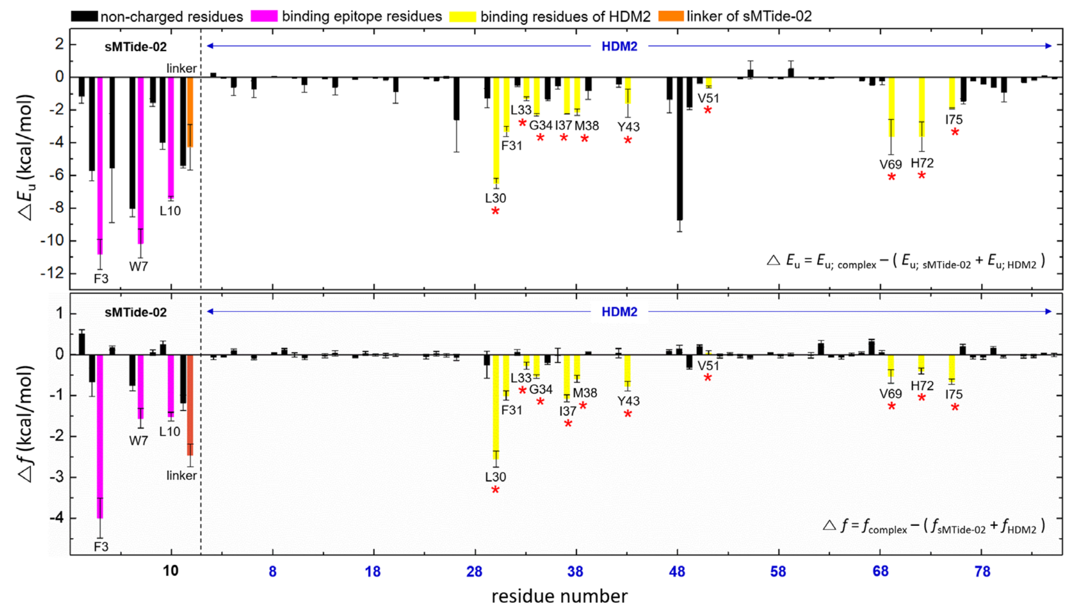

To simplify our argument, we focus on the contributions from the residues and the hydrocarbon linker of the stapled peptide (the left part of the vertical dashed line). Just solely based on the decomposition of $\Delta E_u$ (upper panel), the relevance of the epitope residues (colored magenta) and the hydrocarbon linker (colored orange) is not clear. Indeed, their contributions are comparable to those from the other residues, and in particular, the linker appears to provide only a tiny contribution (the 8th largest contribution).

On the other hand, we obtain drastically different results based on the decomposition of $\Delta f$ (lower panel). The significance of the epitope residues (colored magenta) is apparent. In particular, the relevance of the contribution from the hydrocarbon linker (colored orange) is now evident (the 2nd largest contribution). This reflects the fact that the formation of peptide–protein contacts (resulting in $\Delta E_u < 0$) does not warrant their contributions to the binding affinity because of the associated dehydration penalty ($\Delta G_{solv} > 0$). This demonstrates the essential importance of analyzing $\Delta f = \Delta E_u + \Delta G_{solv}$, rather than examining just $\Delta E_u$, in identifying the residues critical to binding. We are able to perform such an analysis since we have developed our original method for exactly decomposing the solvation free energy (J. Chem. Phys. 2011, 135, 034506, cited as Ref. [20]).

To clearly state these points, we have added the following statements in the revised manuscript, along with a new figure (S1 Fig) showing the residue decomposition of $\Delta f$, $\Delta E_u$ and $\Delta G_{solv}$ (the figure shown above is taken from this new figure).

> (Line 53–60 in Introduction)
> The formation of peptide–protein contacts, such as hydrogen bonds and van der Waals contacts, leads to favorable changes in the direct interaction energy ($\Delta E_u < 0$). However, the formation of these peptide–protein contacts involves the dehydration penalty ($\Delta G_{solv} > 0$). Therefore, in arguing the net contribution to the binding affinity, it is essential to analyze $\Delta f$ that simultaneously takes into account both $\Delta E_u$ and $\Delta G_{solv}$. Importantly, $\Delta f$ can be decomposed into contributions from specific groups constituting the complex [20,21]. Thereby, our method allows us not only to investigate the relevance of individual amino acids, but also to quantify the contribution from the hydrocarbon linker to the binding affinity.
>
> (Line 168–175 in Results and Discussion)
> In this regard, we emphasize the more relevance of analyzing $\Delta f$ than just examining the direct peptide–protein interaction energy ($\Delta E_u$). Indeed, as can be inferred from S1 Fig that further partitions $\Delta f$ into $\Delta E_u$ and $\Delta G_{solv}$ terms, the contributions from the epitope residues and the hydrocarbon linker to $\Delta E_u$ are comparable to those from the other residues in the stapled peptide, and hence, their significance cannot be

elucidated solely in terms of $\Delta E_u$. Only after taking into account the dehydration penalty embodied in $\Delta G_{solv}$, the special role of the epitope residues and the hydrocarbon linker becomes evident. This demonstrates the essential importance of analyzing $\Delta f$ in identifying the residues critical to binding.

We have also added corresponding new figures (S2 and S3 Figs) for the other two systems (formed with sMTide-02b and sMTide-02c) studied in the present work.

**<u>Peer Reviewer #2 (Review Text):</u>**

Comments:
All comments have been addressed. I just note that the resolution of the figures should be improved before publication, and the phrase “major favorite contributor” in the abstract should be reworded for readability.

Author reply:
High resolution figures (tiff files) are uploaded in our resubmission. In response to the reviewer's comment, we have replaced the phase "a major favorite contributor to the binding affinity” with "a sum of the direct peptide–protein interaction energy and the dehydration penalty” in the abstract.
