# Supplementary material for: Effect of linker on the binding free energy of stapled p53/HDM2 complex
Source: PLoS One. 2020 Apr 30;15(4):e0232613. doi: 10.1371/journal.pone.0232613 (PMC7192472; doi:10.1371/journal.pone.0232613)
Supplement: Attachment — Submitted filename: response_letter.docx [file pone.0232613.s004.docx]

Response to Reviewers’ Comments

- We thank the reviewers for their valuable comments and suggestions, which we have incorporated in this revision.
- The changes we made in response to the reviewers’ comments are marked with blue color in an annotated copy of the manuscript, which is uploaded separately.

Peer Reviewer #1 (Review Text):

Comments:

The manuscript closely replicates the work of Thomas et al (Cell Cycle 2010, 9, 4560-4568). Although the authors have cited this particular work, they do not discuss the difference, if any, in their approach and results. The conclusions presented also do not add to our present knowledge, as it is already well known that F19, W23, L26 and the hydrocarbon staple are the most important residues in the binding of stapled p53 peptides to HDM2. The authors claim that their “method will be useful in designing new stapled peptides in which the staple location can be optimized from the thermodynamic viewpoint”. However, they only did simulations of a single stapled peptide and did not compare it with peptides stapled at other positions.

Author reply:

As suggested by the reviewer, we have carried out new simulations with two additional peptides stapled at other positions. The linker positions of the two additional peptides were chosen by referring to the previous experimental work on SAH-p53 (stabilized alpha-helix of p53) peptides. We find that the linker-position dependence of the thermodynamic characteristics is in accord with the experimental observation for the related peptides. These new results are included in the revised manuscript.

Although ten simulations were performed, standard deviations are not shown in Figures 2 and 4.

Author reply:

In response to the reviewer’s comment, we have added standard errors as bars in Figures 2 and 4 of the revised manuscript.

In light of the lack of new results and conclusions, I am hesitant to accept this manuscript for publication.

Author reply:

We hope that, with the inclusion of the new results for the peptides stapled at different positions, our manuscript is acceptable for publication.

Peer Reviewer #2 (Review Text):

Comments:

This manuscript presents the results of molecular dynamics simulation and end-point effective free energy calculations for a stapled peptide interacting with HDM2. The main finding of the paper is that, in addition to the known epitope residues, the hydrocarbon linker imparts a significant contribution to the binding affinity. Overall, the paper meets the standards of publication of PLOS One. Below I have a few comments that, when addressed, would improve the manuscript as well as place it better within the prior literature.

Author reply:

We are glad to hear that our paper meets the standards of publication of PLOS One. We addressed all the comments by the reviewer to further improve our manuscript.

1. As the “effect of linker” is a key area of concentration of this paper, it would be advisable to add some additional calculations and/or comparison of HDM2/peptide interactions where a linker is not present.

Author reply:

We have carried out additional simulations with peptides stapled at other positions, instead of performing calculations for unstapled peptides. We believe that this is also a reasonable way for examining the “effect of linker”. The linker positions of the two additional peptides were chosen by referring to the previous experimental work on SAH-p53 (stabilized alpha-helix of p53) peptides. We find that the linker-position dependence of the thermodynamic characteristics is in accord with the experimental observation for the related peptides. These new results are included in the revised manuscript.

2. More detail describing the effective binding free energy calculation should be added to the abstract, as “binding free energy analysis” encompasses many very different techniques.

Author reply:

In response to this comment, we have added the following statements to the abstract concerning the binding free energy analysis we performed in the present study:

The binding thermodynamics analysis is done based on the end-point calculation of the effective binding energy—a major favorite contributor to the binding affinity—and on its decomposition into contributions from specific groups constituting the complex. This allows us to investigate how individual amino acids in the stapled p53 and HDM2 contribute to the binding affinity.

3. The “Stapled-p53” shorthand that is used throughout can be more clearly defined as the peptide is based upon, but is not, p53. For example, the peptide name used in Ref. 10 for this sequence (sMTide-02) can be utilized.

Author reply:

As suggested by the reviewer, the stapled peptide studied in the present work is referred to as sMTide-02 in the revised manuscript.

4. Simulation work on closely related systems should be cited. There are several works that study binding thermodynamics and kinetics of MDM2/HDM2 and p53-like peptides (see e.g. [1] Zwier, et al J. Phys. Chem. Lett. 2016, 7, 3440. [2] Paul, et al. Nature Comm. 2017, 8 1095, [3] Morrone, et al. J. Chem. Theory Comput. 13 863, [4] Morrone, et al. J. Chem. Theory Comput. 2017 13 870, [5] Tran, et al . J. Phys. Chem. B 2019 123 2469.) Specifically, #3 simulates stapled peptides and finds that the peptide staple may interact with the protein.

Author reply:

We thank the reviewer for letting us be aware of these related works, all of which are cited in the revised manuscript (new references 10–13 and 35).
